# AKI After Hepatic Histotripsy

**DOI:** 10.1016/j.ekir.2026.106603

**Published:** 2026-05-13

**Authors:** Salman Bhutta, Rimda Wanchoo, Vipulbhai Sakhiya, Sepideh Gholami, Craig Devoe, Kenar D. Jhaveri

**Affiliations:** 1 Division of Kidney Diseases and Hypertension, Northwell Health and Donald and Barbara Zucker School of Medicine at Hofstra/Northwell, Great Neck, New York, USA; 2 Department of Surgery and the Northwell Health Cancer Institute, Northwell Health, New Hyde Park, New York, USA; 3 Division of Hematology-Oncology, Northwell Health Cancer Institute, Northwell Health, New Hyde Park, New York, USA

To the Editor:

Histotripsy is a noninvasive focused ultrasound technique that mechanically destroys targeted tissue via acoustic cavitation without thermal injury. In the HOPE4LIVER prospective multicenter trial of 44 patients with liver tumors ≤ 3 cm, adverse events were largely mild (e.g., pain, fatigue, and pyrexia), and acute kidney injury (AKI) was not reported in pivotal or feasibility studies.^1^^,^^2^ Supplementary material summarizes the procedure in detail.

We report a case of AKI following hepatic histotripsy. A 61-year-old male with stage IV colon cancer with liver metastases, diabetes mellitus, and hyperlipidemia had previously undergone colectomy, hepatic interventions, chemotherapy, and radiation. After recurrence, he was restarted on capecitabine. Imaging identified a ∼2.2 cm segment 5 lesion suitable for histotripsy. The procedure was completed under general anesthesia (17.3 cc treated over ∼30 minutes) without complications; no nonsteroidal anti-inflammatory drugs were administered, no hypotension occurred, and blood loss was minimal. Urine output data was not documented.

Baseline serum creatinine was 0.87 mg/dl. Two days post-procedure, serum creatinine rose to 3.24 mg/dl (Kidney Disease: Improving Global Outcomes stage 3 AKI), prompting hospitalization. The patient was asymptomatic and euvolemic, with no nephrotoxic exposures or hemodynamic instability. Urinalysis showed trace proteinuria and microscopic hematuria without casts; protein-to-creatinine ratio was 0.3. Fractional excretion indices suggested intrinsic injury. Serologic workup (antinuclear antibody, antineutrophil cytoplasmic antibodies, and anti-glomerular basement membrane), complement levels, and monoclonal studies were normal. Imaging excluded obstruction, and creatine kinase was normal. With i.v. fluids, serum creatinine improved to 2.66 mg/dl within 24 hours and normalized to 1.06 mg/dl within 10 days, with resolution of hematuria. Supplementary material has additional detailed test results and information about the case.

Postmarketing, the Food and Drug Administration Manufacturer and User Facility Device Experience database review identified 8 additional cases of renal impairment after hepatic histotripsy, typically in females (age 57–70) and often with larger treatment volumes (> 90 cc) (Table 1). Two required dialysis and 2 deaths were reported; others recovered with hydration.^3^^,^^4^ In all the other cases, the AKI resolved with hydration. One major limitation of the Manufacturer and User Facility Device Experience database is the lack of detailed information and long-term data. In response to these real-world reports, device labeling has been updated to include a warning that multiple or large-volume liver treatments have been associated with AKI and to emphasize appropriate clinical monitoring.^5^

**Table 1:** Summary of the cases reported thus far with histotripsy associated kidney injury

Case #	Source	Age	Sex	Cancer type/treatment volume	Reported event	Peak serum creatinine (mg/dl)	Outcome	Dialysis (yes/no)	Device malfunction
1	FDA^3^	68	Female	Mets-Neuroendocrine tumor 50.6 cc	AKI, hospitalization	Not reported	Normalized with hydration discharged	No	No
2	FDA	61	Female	Mets-Neuroendocrine tumor 109.6 cc	AKI, anuria, ICU stay, fluids, hospitalization	Not reported	Normalized with hydration, discharged	No	No
3	FDA	71	Female	Mets-ovarian cancer 53 cc	AKI, second degree burn, dark urine, blood, hospitalization	2.1	Normalized with hydration, discharged	No	No
4	FDA	74	Female	Mets-colon cancer 111 cc	AKI-D, gall bladder injury, hospitalization, anuric	2.78	Initiated dialysis and last mention, still on dialysis	Yes	No
5	FDA	79	Female	Gall bladder cancer 96.38 cc	Hypotension, septic shock, AKI, hematuria, proteinuria	Not reported	No nephrotic syndrome, patient was made comfort measure- death	No	No
6	FDA	76	Female	Mets-colon cancer 101.1 cc	AKI- persistent following post-procedural hemorrhage and s/p embolization of a subsegmental inferior left hepatic artery branch. Presumed ATN given granular casts noted	Not reported	Did not recover- death	No	No
7	FDA	63	Female	Mets-breast cancer 114 cc	AKI-D, hyperbilirubinemia, got fluids	7	Did not recover- required dialysis on discharge	Yes	No
8	Reference # 4	58	Female	Mets- neuroendocrine tumor	AKI, needed fluids diuretics	Not reported	Normalized	No	No
9	Our case	61	Male	Mets-colon cancer 17.3 cc	AKI, hematuria, hospitalized	3.24	Normalized with hydration	No	No

AKI, acute kidney injury; ATN, acute tubular injury; D, dialysis; FDA, Food and Drug Administration; ICU,- intensive care unit.

Potential mechanisms include pigment-mediated tubular injury from hemolysis and release of cellular debris, as well as hemodynamic or immune-mediated interstitial injury. Preventive strategies such as ensuring adequate pre- and postprocedure hydration, close laboratory monitoring, and minimizing exposure to additional nephrotoxic insults, including contrast-based imaging when feasible, may help mitigate this risk. Further study, including biopsy in select cases, is needed to clarify pathophysiology and risk mitigation.

## Disclosure

KDJ serves as a consultant for Novartis, Amgen, Calliditas, Travere, Otsuka, Emerald Clinicals, Apellis and is a contributor to Uptodate.com and Lexicomp. He also serves as Editor-in-Chief for ASN Kidney News. SG serves as a consultant for DelCath, Iota, and Alphasight; an institutional principal investigator for clinical trials sponsored by AstraZeneca; and is an educator for HistoSonics. RW serves as consultant for Alexion. All other authors have no conflict of interests to disclose.

## Patient Consent

The patient consent was obtained.
