# Supplementary material for: AKI After Hepatic Histotripsy
Source: Kidney Int Rep. 2026 May 13;11(7):106603. doi: 10.1016/j.ekir.2026.106603 (PMC13254590; doi:10.1016/j.ekir.2026.106603)
Supplement: Supplementary File (PDF) [file mmc1.pdf]

Acute Kidney Injury Following Hepatic Histotripsy- Supplemental Material

Salman Bhutta[1], Rimda Wanchoo[1], Vipulbhai Sakhiya[1], Sepideh Gholami[2], Craig Devoe [3] & Kenar D Jhaveri [1]

[1]Division of Kidney Diseases and Hypertension and [3]Division of Hematology-Oncology and Northwell Cancer Institute, NY, USA and [2]Department of Surgery, Northwell Health, New Hyde Park, NY, USA

1. What is Histotripsy?

Histotripsy is a non-invasive, non-thermal ablation technique that uses focused high-intensity ultrasound to mechanically destroy targeted tissue via acoustic cavitation. The HistoSonics Edison® system has been evaluated clinically for the treatment of primary and metastatic liver tumors and received regulatory clearance based on safety and efficacy endpoints.

In the procedure suite (interventional radiology or hybrid OR), patients typically undergo general anesthesia to ensure immobility and precise targeting. Standard pre-medications include induction agents (e.g., propofol, opioids), antiemetics such as Ondansetron, and selective use of antibiotics depending on case complexity and infection risk. In some cases, maintenance IV fluids (normal saline or lactated Ringer's) are administered to maintain euvolemia and hemodynamic stability throughout the procedure.

After positioning and application of an acoustic coupling medium, real-time ultrasound imaging is used to localize the lesion and map a treatment plan. A focused ultrasound transducer then delivers high-amplitude pulses to generate cavitation bubble clouds, which rapidly expand and collapse, mechanically fragmenting cells without thermal injury. The operator systematically moves the focal point across the lesion in a raster pattern, treating small volumes sequentially while monitoring cavitation activity in real time.

Once complete, imaging confirms adequate coverage. No incisions or probes are required, recovery is rapid, and the treated tissue is gradually resorbed.

2. Additional Case Details

The patient mentioned in the report had undergone a left colectomy, hepatic resection/ablation, and perioperative chemotherapy with modified leucovorin, 5-Fluorouracil, Irinotecan and Oxaliplatin. Stereotactic radiation was administered for recurrence in the ablation cavity, followed by panitumumab and capecitabine up to 6 months prior to this presentation. A few months prior to AKI, MRI showed interval growth of a segment 5 hepatic lesion and a new segment 7 lesion. He was restarted on capecitabine monotherapy. A screening ultrasound identified a ~2.2 cm segment 5 liver lesion suitable for histotripsy.

The patient underwent successful histotripsy under general anesthesia (17.3 cc treated over ~30 minutes) with no complications, no NSAIDs administered, no

hypotensive episodes, minimal blood loss, and stable post-procedure status. His urine output was not decreased.

His lab parameter details:

Urinalysis showed trace proteinuria, moderate blood, and 8 red blood cells per high power field with no urinary casts. The urine protein-to-creatinine ratio was mildly elevated at 0.3. His urine sodium was 47 meq/L, urine potassium: 23.9 meq/L with an urinary Na/K ratio: 1.97. No prior urinalysis was available within the preceding 2 years.

His fractional excretion of Na was 1.4%.

His fractional excretion of urea was 49.3%.

A kidney sonogram showed no evidence of hydronephrosis.

Hemolysis lab parameters were not checked.

Cystatin C based GFR was not assessed.

Creatinine kinase level was in normal range.

Serologic evaluation was unrevealing (normal ANA, ANCA and anti GBM titers), his serum free light chain ratio was 1.3 and normal serum immunofixation, C3 and C4 in normal range.

There was no evidence of anemia.
